# Successful Resolution of Chronic Testicular Pain With an Impairment-Based Treatment Program: A Case Study With One-Year Follow-Up

**DOI:** 10.7759/cureus.13850

**Published:** 2021-03-12

**Authors:** David P Newman, Nicholas H Tinkham, Joseph R Sterbis, Adam T Soto

**Affiliations:** 1 Pain Management, Tripler Army Medical Center, Honolulu, USA; 2 Urology Service, Tripler Army Medical Center, Honolulu, USA

**Keywords:** orchialgia, testicular pain, physical therapy, genitofemoral nerve, ilioinguinal nerve, manual therapy, urology, soft tissue mobilization

## Abstract

Chronic testicular pain is a condition commonly experienced by males. Potential causes of testicular pain can be pathology localized within the testicle or referred pain from surrounding tissues or spinal conditions. The diagnostic differential is extensive and can be seen as a diagnosis of exclusion after structural disorders specific to the testicle are ruled out. In approximately 50% of the cases, the cause of pain is undetermined. Patients with testicular and inguinal pain may undergo extensive workup that overlooks potential neuropathic and musculoskeletal causes remote to the testicle. This case study describes the application of a conservative treatment program targeting presumptive chronic genitofemoral and/or ilioinguinal nerve entrapment along the course of the inguinal canal for the treatment of chronic testicular pain. By combining sacroiliac joint osteopathic manipulation, iliopsoas stretching, and soft tissue mobilization utilizing a vacuum suction cup, the patient was symptom-free on the fourth visit after suffering from testicular pain for a year. At a one-year follow-up, the patient remains pain-free.

## Introduction

Chronic testicular pain, or orchialgia, is defined as intermittent scrotal pain of a three-month duration that significantly affects the patient’s function [1]. While patients primarily experience testicular pain, pain external to the testicle can be noted along the spermatic cord, groin, suprapubic region, flank, abdomen, and perineum [2]. Approximately 100,000 men per year experience testicular and groin pain [3]. In up to 50% of cases, the exact cause of chronic testicular pain is undetermined [4]. Potential causes include structural disorders (i.e. spermatocele, varicocele, trauma, epididymitis, and tumor), trauma, sports hernia, low back injury, post-vasectomy syndrome, prostatitis, pelvic floor dysfunction, and nerve entrapment [3,5-8].

In cases where nerve entrapment is suspected, several factors must be considered. Idiopathic ilioinguinal nerve entrapment is rare [6]. Genitofemoral and ilioinguinal nerve entrapment or injury is more common following herniorrhaphy [8]. Finally, physical compression of the nerve(s) may not be appreciated upon imaging, but instead, observed during surgical exploration [7].

There appear to be three different pathoanatomic mechanisms for genitofemoral, ilioinguinal, and/or iliohypogastric nerve entrapment. First, an imbalance between the abdominal and hip adductor muscles results in a weakening of the posterior wall of the inguinal canal [9]. This may result in bulging of the abdominal structures, thereby compressing the genitofemoral and ilioinguinal nerves [7,9]. Second, there are congenital variations in the course of these nerves and tissue adhesions linked to previous inflammation, surgery, or other traumatic and metabolic conditions that may not allow the nerves to stretch when stressed during activity [7]. Finally, idiopathic entrapment may occur when the transverse abdominus or internal oblique muscles contract resulting in mechanical irritation of the ilioinguinal nerve due to its passage through those muscles [5,10]. The ilioinguinal nerve pierces both abdominal muscles prior to entering the inguinal canal [6]. In a study involving 51 athletes with groin pain recalcitrant to conservative measures, surgical exploration and neurolysis were performed [7]. Ninety-six percent of the patients demonstrated nerve entrapment, primarily the ilioinguinal nerve (92.5%) due to nerve adherence to surrounding soft tissue. In 79.3% of the cases, posterior inguinal wall attenuation was seen.

The purpose of this case study is to describe the application of osteopathic manipulation, instrument-assisted deep tissue mobilization, and a single targeted stretch for a patient with chronic inguinal and testicular pain and propose a novel impairment-based treatment program that may be utilized in future studies.

## Case presentation

A 27-year-old male medical technician presented with a 12-month history of right-sided inguinal and testicular pain. The patient’s symptoms were insidious in onset with no history of trauma, injuries related to sports or work activities, medications used to account for the symptoms or familial history of genitourinary conditions. At the time of initial occurrence, the patient was seen in the primary care clinic with a complaint of painful urination with bilateral suprapubic pain, bilateral flank pain, and back pain. Pain severity ranged from 6/10 to 8/10 on a visual analogue pain scale. No hematuria or testicular symptoms were present. A microscopic urinalysis and abdominal and pelvis computed tomography (CT) scan were ordered to rule out nephrolithiasis and potential inguinal hernia. The urinalysis was normal. While no nephrolithiasis or obstructive uropathy was identified on the CT scan, a left-sided prostatic calcification was noted. The initial treatment consisted of rest and indomethacin.

Symptoms did not improve over one week and the patient returned to his physician. A urinalysis was obtained and showed increased nitrites and protein suggesting a urinary tract infection, which was empirically treated with sulfamethoxazole and trimethoprim for three days. While there was a mild improvement initially, the symptoms reoccurred. Based on a repeat CT finding and an International prostate symptom score of 25, the differential diagnosis seemed to favor prostatitis. Over the next six weeks, the working diagnosis evolved to include pelvic pain syndrome. Further, urology and gastroenterology workup were performed and found to be non-contributory for a definitive diagnosis. The patient underwent a right cord nerve block targeting the genital branch of the genitofemoral nerve and the ilioinguinal nerve. By two days following the injection, the patient reported an increase in testicular pain at the injection site and worsening pain when the testicle was not supported. He reported waking one to two times per night due to pain.

The patient underwent a five-week course of pelvic floor physical therapy. Treatment consisted of pelvic floor and abdominal muscle retraining utilizing biofeedback. Biofeedback-based pelvic floor muscle re-education has been shown to be effective in treating men with chronic prostatitis and chronic pelvic pain syndrome [11]. Upon discharge, the patient reported improvements in his hip flexibility and urination frequency. However, there were no long-term improvements in pain and the patient continued to have pain that limited his physical activities, marital relationship, and sleep. The patient was not satisfied that all treatment options had been exhausted. He was subsequently referred to the Interdisciplinary Pain Management Clinic (IPMC) for evaluation and management. Upon presentation, the patient’s goals were diagnostic clarity, avoiding interventional injections, and resolution of symptoms.

Physical examination/evaluation

**Figure 1:**
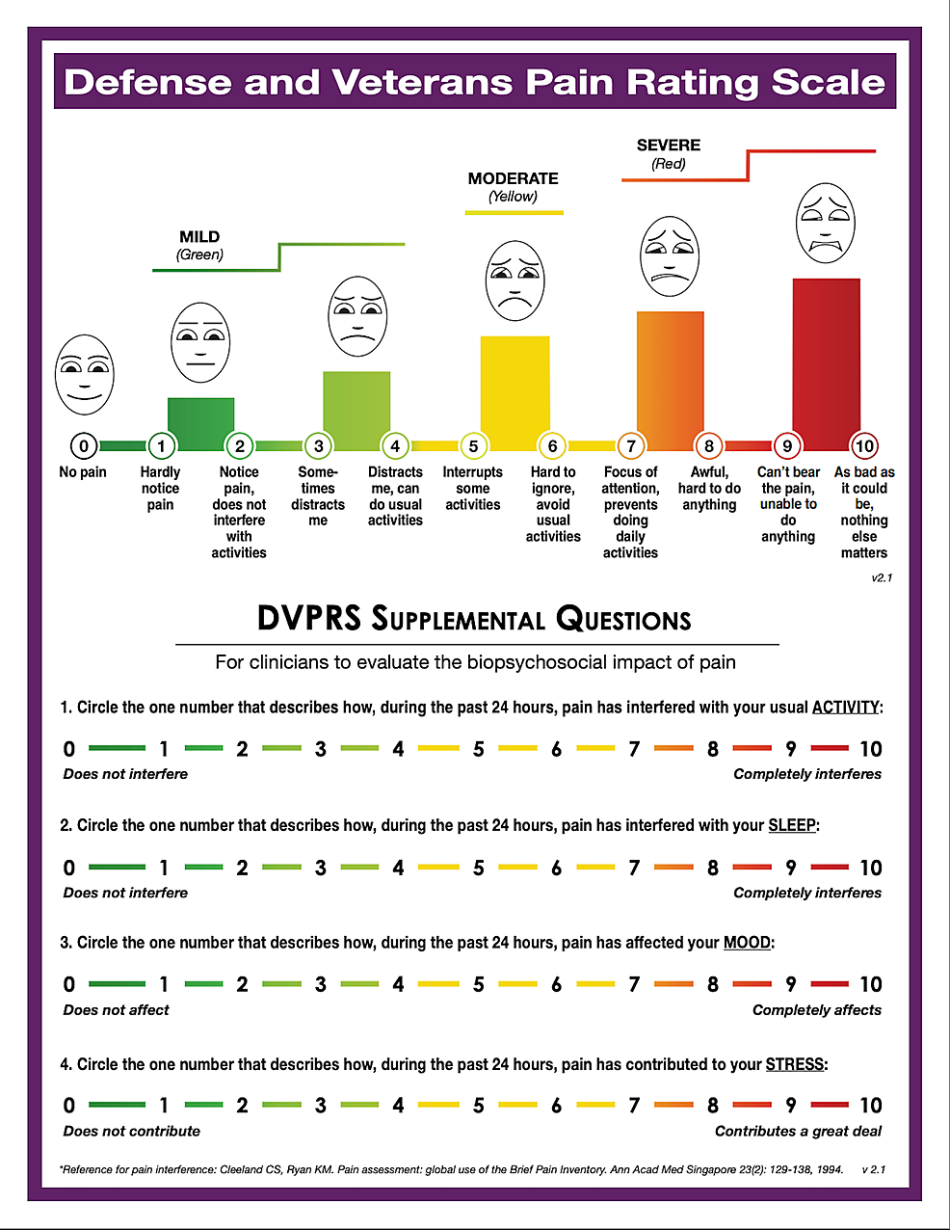
Defense and Veterans Pain Rating Scale (DVPRS)

Physical evaluation by the IPMC physical therapist revealed that the patient’s pain was localized to the right inguinal area and testicle. Digital pressure applied distal to the inguinal canal at the external inguinal ring resulted in both inguinal and the testicular pain, consistent with his index pain. This finding is seen in approximately 94% of patients with a sports hernia and tenderness with palpation is part of the diagnostic criteria for the inguinal-related groin pain classification per the Doha agreement [12,13]. The patient completed a Defense and Veterans Pain Rating Scale (DVPRS) (Figure 1) with his pain level rated at 1/10 [14]. The subsets of the pain supplemental questions for activity, sleep, mood, and stress were rated at the initial encounter as 2/10, 2/10, 3/10, and 3/10, respectively, and were assessed at each follow-up visit (Table 1). The patient did not report any pain along the lumbar spine. The patient demonstrated a full lumbar range of motion, but he described a pulling-type sensation along the groin with end range extension. The right hip extension was decreased compared to the left side. Posterior anterior pressure applied to the spinous processes of L1 through L5 into the end range of joint motion did not change his pain.

**Table 1:** DVPRS per Visit DVPRS - Defense and Veterans Pain Rating Scale

	First visit	Second visit	Third visit	Fourth visit	Final visit
Current Pain Level	1	3	2	0	0
Pain With Activity	2	2	2	0	0
Pain Affecting Sleep	2	2	1	0	0
Pain Affecting Mood	3	3	2	0	0
Pain Contributed to Stress	3	3	2	0	0

Sacroiliac joint (SIJ) motion tests

In standing, the physical evaluation revealed asymmetry in the patient’s pelvic landmarks with his posterior superior iliac spine (PSIS) and iliac crest elevated on the right side as compared to the left. The SIJ motion tests utilized were the forward flexion test and the thigh thrust test. In the forward flexion test, the PSIS’s are assessed for motion as the patient bends forward from a standing position. The response is positive if the PSIS moves first and/or is higher on the painful side. For the thigh thrust, the patient is supine with hip and knee flexed. The examiner cups the sacrum with one hand and applies force axially through the knee providing a shear force to the SIJ. The test is positive if the pain is reproduced in the SIJ region. In this case, both tests were positive demonstrating right-sided SIJ hypomobility.

Strength and flexibility assessment

During the initial evaluation, the patient’s flexibility was also assessed. The patient presented with the tightness of the right iliopsoas. Manual resistance was then applied to the iliopsoas reproducing the patient’s pain mildly. This evaluative technique is recommended by Knockaert, D’Heygere, and Bobbaers as a maneuver to diagnose ilioinguinal nerve entrapment [6].

Diagnosis/prognosis

Differential diagnosis specific to this patient’s symptoms upon initial evaluation to the IPMC was extensive and included chronic testicular pain, sports hernia, neuropathic pain, genitofemoral nerve entrapment, ilioinguinal nerve entrapment, and abdominal cutaneous nerve entrapment syndrome (ACNES). As symptoms upon onset were painful urination and bilateral suprapubic pain, ACNES was not considered. The spermatic cord block increased the patient’s pain; therefore, nerve entrapment within the scrotum and varicocele were discarded. Similarly, the specific diagnosis of sports hernia was low on the differential due to a lack of trauma or activity-related injury; however, ilioinguinal nerve and the genital branch of the genitofemoral nerve entrapment are pathoanatomic components of a sports hernia [12]. Instead, tissue-based nerve entrapment proximal to the scrotum was the likely mechanism since the patient’s testicular pain was reproduced with deep pressure applied to the superficial inguinal rings, a mild reproduction of symptoms with the loading of the iliopsoas, and no benefit from the scrotal block.

The prognosis for full resolution of symptoms was poor given the chronicity of symptoms, the poor response to conservative measures, and the patient’s request to avoid interventional procedures. While the pain levels reported would appear comparatively low, the patient’s behavioral health and marital relationship challenges were an overriding factor in proceeding with care.

Intervention

The plan of care included manual therapy, stretching, strengthening, instrument-assisted tissue mobilization (IASTM), and activity modification (Table 2). The impairment-based rehabilitation program was designed to leverage osteopathic manipulation techniques (OMT) and IASTM in the clinic with a self-management program.

**Table 2:** Overview of Interventions Applied and Patient Response per Visit

Visit	Patient Pain Presentation	Objective Findings	Intervention	Patient Response
1	Pain localized to the right inguinal area and testicle.	Pain reproduced with digital pressure applied to the external inguinal ring. SIJ hypomobility. Pain reproduced with manual resistance applied to the iliopsoas.	Right SIJ OMT. Manual stretching of the right iliopsoas muscle.	No change in pain with SIJ OMT. Mild decrease in pain with digital pressure at the external inguinal ring after stretching.
2	An increase in inguinal and testicular pain since the initial visit.	Negative SIJ exam. Pain reproduced with digital pressure applied to the external inguinal ring.	Directional cupping applied to the length of the inguinal canal.	Post treatment soreness.
3	A decrease in inguinal and testicular pain at presentation.	Pain reproduced with digital pressure applied to the external inguinal ring.	Directional cupping applied to the length of the inguinal canal. Manual iliopsoas stretching.	Post treatment soreness. No increase in pain with digital pressure at the external inguinal ring.
4	No complaint of inguinal or testicular pain.	No pain reproduced with digital pressure applied to the external inguinal ring or along the length of the canal. No pain reproduced with lunging, squatting, or eccentric sit-ups. Mild tightness of the iliopsoas without pain.	Active release technique applied to the iliacus. Manual stretching of the iliopsoas.	No pain reproduced with digital pressure at the external inguinal ring.
5	No complaint of inguinal or testicular pain.	Benign physical exam except mild tightness of the right iliopsoas compared to the left side.	Active release technique applied to the iliacus. Manual stretching of the iliopsoas. Discharge from IPMC.	

**Figure 2:**
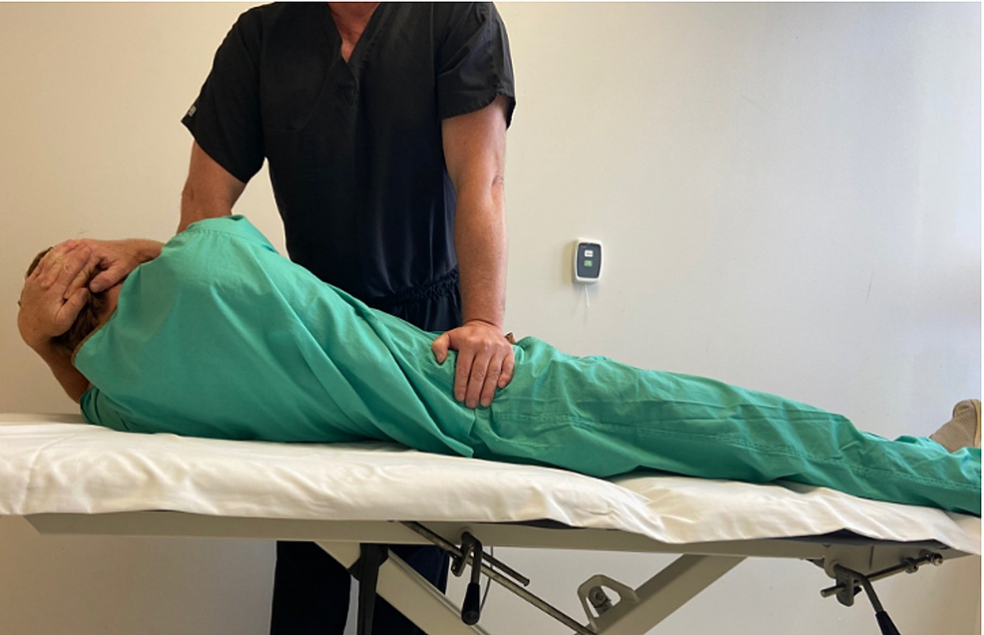
Sacroiliac Joint Manipulation Technique With the patient in supine, the provider passively side bends the patient to the right and then rotates the trunk to the left locking down the lumbar spine. The provider pushes the right ilium into a posterior direction until a barrier is felt. As the patient exhales, the provider imparts a high velocity, low amplitude force downwards. (Photograph: Newman, DP. Sacroiliac Joint Manipulation Technique. Reproduced with permission of author, 2021).

**Figure 3:**
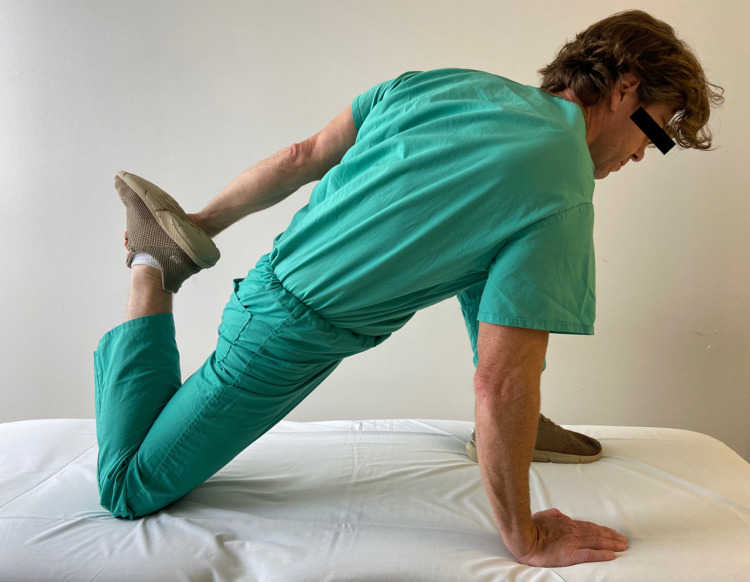
Iliopsoas Stretch Demonstration of the right iliopsoas being stretched. Additional muscles being affected are the quadriceps as the rectus femoris is a hip flexor. (Photograph: Newman, DP. Iliopsoas Stretch).

Upon initial treatment, the patient underwent a common OMT directed at the right ilium to address the SIJ dysfunction (Figure 2). This maneuver did correct the SIJ mechanics, but there was no change in pain symptoms with digital palpation at the external inguinal ring. Manual stretching was then applied to the right iliopsoas for 30 seconds per repetition over a 10-minute period. There was a mild decrease in pain with pressure applied to the external inguinal ring. The patient was started on a home exercise program consisting of iliopsoas stretching (Figure 3). This stretch was selected to reduce the anterior translation of the ilium, which could have counteracted potential benefit from the OMT maneuver's posterior translation of the ilium. The patient was asked to follow-up seven days later.

**Figure 4:**
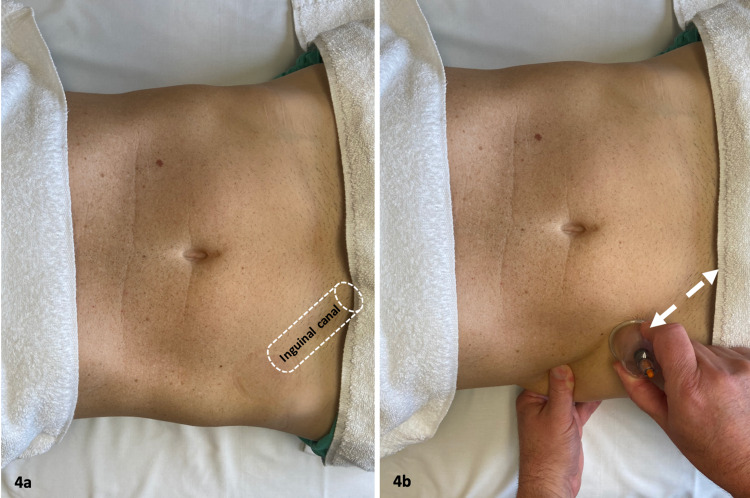
Directional Cupping Technique. (a) The inguinal canal is identified. (b) The cup placement and area treated. After lubricating the skin with lotion, the 4.0 cm diameter vacuum suction cup (KangZhu, China) was applied. The vacuum suction cup was manually moved across the skin with the goal of mobilizing the soft tissue along the course of the inguinal canal to the superficial inguinal ring. This was repeated several times, taking breaks when the discomfort was too intense. The technique was performed for a total period of one minute. (Photograph: Newman, DP. Directional Cupping Technique. Reproduced with permission of author, 2021).

At the first follow-up, the patient reported that the stretching program only provided short-term benefit. His pain level was a 3/10, which represented a two-point increase in his baseline pain level. The SIJ exam was negative. Testicular pain was again reproduced with digital pressure applied at the area of the external inguinal ring. The patient was then treated with directional cupping (Figures 4a, 4b) along the length of the inguinal canal from the anterior superior iliac spine (ASIS) to the external inguinal ring so that all potential entrapment areas were addressed [5,7,10]. To determine if this treatment was effective, the patient was asked to strength train at the gym prior to his next visit.

By the third visit three days later, the patient reported a small improvement in pain; however, there continued to be a 2/10 pain level after working out. The directional cupping treatment was repeated followed by manual iliopsoas stretching. The patient was placed on the left side lying position and pulled the left knee towards the chest. The provider held the right leg with knee flexed to 90 degrees, and moved the leg posteriorly until a stretch was felt. The stretch was held for 30 seconds and repeated five times. The patient was asked to follow-up in one week.

At the fourth visit, the patient reported no pain since the previous treatment session. The patient returned to pain-free jogging and engaged in intercourse without pain. The patient reported that he consistently stretched the iliopsoas. Upon examination, no tenderness or radiating pain was reproduced with digital pressure over the external inguinal ring. The patient performed exercises that would have previously exacerbated his pain to include squatting, lunging, and eccentric sit-ups. No pain was reproduced. The patient was asked to continue his stretching program to equalized iliopsoas length as compared to the opposite side and to follow-up as needed if any return of pain. He followed-up two days later wanting to undergo another treatment to stay ahead of re-injury. The patient reported no pain across all DVPRS subsets (Table 1). The final intervention provided was an active release technique to inhibit the iliopsoas and compliment manual stretching. He was encouraged to return to the clinic for any recurrence in inguinal or testicular pain.

The patient was seen in the IPMC for an unrelated musculoskeletal complaint one-year following completion of therapy for this inguinal pain. He reported one episode of inguinal pain during this period that resolved after stretching of the iliopsoas and performing deep tissue massage along the inguinal canal utilizing a Thera Cane (Thera Cane Company, Denver, CO).

## Discussion

In patients presenting with inguinal and testicular pain, it can be difficult to accurately identify a specific diagnosis or pain generator when the diagnostic workup proves non-contributory or provides incidental findings. When testing is negative for a source and there is no anatomic abnormality such as a spermatocele, a urologist’s treatment options are limited. Cord blocks may be utilized to interrupt the afferent pathway thereby relieving pain. Cord blocks have demonstrated short-term relief in over 90% of patients; however, the utility of such procedures may be more diagnostic prior to microscopic denervation of the spermatic cord [15]. In our case, the cord block increased the patient’s pain suggesting an extra-scrotal source of pain or central sensitization [15].

Patients referred to Pain Management may benefit from several treatment options including pharmacologic and procedural modalities. Pharmacologic treatments will vary based on the pain pathology, namely nociceptive vs. somatic/visceral vs. neuropathic, and often involve NSAIDs followed by neuropathic medications including anticonvulsants and antidepressants [15]. Anticonvulsants typically include calcium channel blockers such as gabapentin or pregabalin. Antidepressants include tricyclic antidepressants, such as amitriptyline or nortriptyline, and serotonin-norepinephrine reuptake inhibitors, including duloxetine and venlafaxine.

Procedural treatments include nerve blocks of the genitofemoral and/or ilioinguinal nerves, pulsed radiofrequency ablation (RFA) or cryoablation of these nerves, or a spinal cord stimulator (SCS). Hetta et al. published a double-blind randomized controlled trial (RCT) reporting greater than 50% improvement in chronic post-surgical testicular pain at 3 months following pulsed RFA of both the ilioinguinal and genitofemoral nerves [16]. While an SCS is a potential treatment option, there has only been case reports describing its efficacy [17].

Conservative management of patients with chronic inguinal and testicular pain due to potential myofascial entrapment of the genitofemoral and/or ilioinguinal nerves can be provided via a multi-modal rehabilitation program utilizing a novel impairment-based treatment program. This program attempts to re-establish the mechanical relationship of the SIJ, lengthen the iliopsoas muscle, and mobilize the soft tissue along the inguinal canal to the external inguinal ring. This case study demonstrates the successful application of this program in a patient with chronic inguinal and testicular pain. At the completion of a three-week program, the patient was pain-free. At one-year following discharge, the patient reported only one episode of inguinal pain which he successfully self-managed with stretching and instrument-assisted, deep tissue mobilization along the inguinal canal.

Upon initial examination by the pain management physical therapist, SIJ hypomobility was noted on the painful side. While there was no change in pain following OMT to the SIJ, this treatment may have been a critical component to the overall outcome. There is significant heterogeneity in pain patterns related to SIJ dysfunction. Following diagnostic SIJ intra-articular injections, 14% of patients reported a decrease in their groin pain [18]. Given the flank and low back pain upon initial presentation to primary care, sacroiliac dysfunction may have been present and contributed to the symptoms. While the diagnostic work-up process to determine if his pain was due to a hernia, nephrolithiasis, or UTI was taking place, a concomitant low risk referral to a musculoskeletal specialist may have been beneficial in reducing potentially unnecessary, costly, and invasive procedures while expediting access to definitive care. 

Iliopsoas stretching was selected given the course of the genitofemoral and ilioinguinal nerves and response to active contraction of the muscle. The genitofemoral nerve penetrates and runs along the anterior aspect of the psoas major before splitting into the genital and femoral branches [19]. The ilioinguinal nerve emerges from the lateral border to the psoas major. While there are no published studies describing either of the nerves being entrapped due to the psoas major muscle tightness, inguinal pain was reproduced when resistance was applied during muscle strength assessment [6]. Inclusion of the muscle stretching in the treatment program was validated when the patient did report a short-term reduction in pain after stretching.

Neural and soft tissue mobilization are common treatment techniques utilized in the management of nerve entrapments [20]. Directional or moving cupping was selected as a treatment modality as traditional soft tissue mobilization techniques require the application of pressure over the nerve, thereby provoking pain. Directional cupping was applied along the course of the inguinal canal as both the ilioinguinal nerve and the genital branch of the genitofemoral nerve run along the inguinal canal passing through the external inguinal ring [19]. The cupping technique extended to the area of the external inguinal ring since both inguinal and testicular pain were reproduced with pressure applied to this area. If this technique were the definitive treatment to reduce compression of the nerve(s), this case would serve to validate tissue-based nerve entrapment. Potential studies to elucidate this pathology may include a sonographic examination of the inguinal area before or after cupping. Case-controlled or randomized controlled studies incorporating the proposed program or cupping alone may also provide more information.

This case study does have several limitations. Inherent in case studies, it is difficult to draw a direct cause and effect as there was no control for comparison. Also, it is difficult to generalize the results across a broad age range and time spectrum. There was a significant delay in referral to both the pelvic floor and pain physical therapist. Future research should consider the impact of this proposed treatment program in patients with acute and chronic conditions.

## Conclusions

Chronic testicular pain is a debilitating condition where it is often difficult to accurately diagnose a discrete pain generator and provide a definite treatment. Patients without pathology specific to the testicle undergo a prolonged and fruitless diagnostic workup. Musculoskeletal and neuropathic causes of pain, specifically genitofemoral and ilioinguinal nerve entrapment, may be underdiagnosed and treated. This case study proposes mechanisms of entrapment and a conservative, multimodal approach for the successful treatment of chronic testicular pain.
